# Trends in hepatocellular carcinoma incident cases in Japan between 1996 and 2019

**DOI:** 10.1038/s41598-022-05444-z

**Published:** 2022-01-27

**Authors:** Masahito Nakano, Hiroshi Yatsuhashi, Shigemune Bekki, Yuko Takami, Yasuhito Tanaka, Yoko Yoshimaru, Koichi Honda, Yasuji Komorizono, Masaru Harada, Michihiko Shibata, Shotaro Sakisaka, Satoshi Shakado, Kenji Nagata, Tomoharu Yoshizumi, Shinji Itoh, Tetsuro Sohda, Satoshi Oeda, Kazuhiko Nakao, Ryu Sasaki, Tsutomu Yamashita, Akio Ido, Seiichi Mawatari, Makoto Nakamuta, Yoshifusa Aratake, Shuichi Matsumoto, Tatsuji Maeshiro, Takashi Goto, Takuji Torimura

**Affiliations:** 1 Division of Gastroenterology, Department of Medicine, Kurume University School of Medicine, Kurume, Japan; 2 Clinical Research Center, National Hospital Organization Nagasaki Medical Center, Department of Hepatology, Nagasaki University Graduate School of Biomedical Sciences, 2-1001-1 Kubara, Omura, Nagasaki 856-8562 Japan; 3 Department of Hepato-Biliary-Pancreatic Surgery, National Hospital Organization Kyushu Medical Center, Fukuoka, Japan; 4 Department of Gastroenterology and Hepatology, Faculty of Life Sciences, Kumamoto University, Kumamoto, Japan; 5 Department of Gastroenterology, Faculty of Medicine, Oita University, Yufu, Japan; 6 Department of Hepatology, Nanpuh Hospital, Kagoshima, Japan; 7 Third Department of Internal Medicine, School of Medicine, University of Occupational and Environmental Health, Kitakyushu, Japan; 8 Department of Gastroenterology and Medicine, Faculty of Medicine, Fukuoka University, Fukuoka, Japan; 9 Division of Gastroenterology and Hematology, Department of Internal Medicine, Faculty of Medicine, University of Miyazaki, Miyazaki, Japan; 10 Department of Surgery and Science, Graduate School of Medical Sciences, Kyushu University, Fukuoka, Japan; 11 Department of Hepatology, Japanese Red Cross Fukuoka Hospital, Fukuoka, Japan; 12 Liver Center, Saga University Hospital, Saga, Japan; 13 Department of Gastroenterology and Hepatology, Nagasaki University Hospital, Nagasaki, Japan; 14 Department of Gastroenterology, National Hospital Organization Oita Medical Center, Oita, Japan; 15 Digestive and Lifestyle Diseases, Kagoshima University Graduate School of Medical and Dental Sciences, Kagoshima, Japan; 16 Department of Gastroenterology, National Hospital Organization Kyushu Medical Center, Fukuoka, Japan; 17 Department of Gastroenterology, Fukuoka Tokushukai Hospital, Kasuga, Japan; 18 First Department of Internal Medicine, University of the Ryukyus Hospital, Nakagami, Japan; 19 Department of Gastroenterology, Nagasaki Rosai Hospital, Sasebo, Japan

**Keywords:** Hepatology, Cancer

## Abstract

We examined the epidemiological trends, including the distribution of sex, age, and disease etiology, in HCC incident cases, over 24 years. Data of 20,547 HCC patients (1996–2019) were analyzed in this prospective study. We divided the study period into four 6-yearly quarters. HCC etiology was categorized as hepatitis B virus (HBV) infection, HBV + hepatitis C virus (HCV) infection, HCV infection, and both negative (non-BC). The incident cases of HCC per quarter of the study period were 4311 (21.0%), 5505 (26.8%), 5776 (28.1%), and 4955 (24.1%), sequentially. Overall, 14,020 (68.2%) patients were male. The number of HCC cases in patients < 60 years, 60–69 years, 70–79 years, and ≥ 80 years were 3711 (18.1%), 6652 (32.4%), 7448 (36.2%), and 2736 (13.3%), respectively. The average age of newly-diagnosed patients increased in each quarter. HCC was associated with HBV, HBV + HCV, and HCV infections and non-BC in 2997 (14.6%), 187 (0.9%), and 12,019 (58.5%), and 5344 (26.0%) cases, respectively. The number of HCV-associated cases decreased in each quarter, while that of non-BC-associated cases increased. HCC incident cases tend to increase in the elderly and in non-BC patients; in contrast, HCC incident cases due to HCV tend to decrease.

## Introduction

In 2018, liver cancer was the sixth most commonly diagnosed cancer and the fourth leading cause of cancer-related deaths worldwide, following lung, colorectal, and stomach cancers, with an estimated 841,000 new cases and 782,000 deaths annually^1–4^. Primary liver cancer includes hepatocellular carcinoma (HCC, comprising 75–85% of cases) and intrahepatic cholangiocarcinoma (comprising 10–15% of cases), as well as some rare disease types^1,2^. The main risk factors for HCC, are chronic infection with hepatitis B virus (HBV) or hepatitis C virus (HCV), high alcohol intake, obesity, and type 2 diabetes^1,2,5–10^.

Globally, HBV infection is the leading cause of incident liver cancer and associated mortality, followed by alcohol consumption, HCV infection, and other causes, which account for 33%, 30%, 21%, and 16% of the total burden of this disease, respectively^2^. The major risk factors associated with HCC vary between regions. In areas considered “high-risk” for HCC, for example, China and East Africa, the critical disease determinant is chronic HBV infection; in contrast, in countries such as Egypt or Japan, HCV infection is likely the predominant cause^1,2^. Recent developments in HCV treatment suggest that a large proportion of liver cancer cases can be prevented^1,11,12^. An interferon (IFN)-free direct-acting antiviral agent (DAA, daclatasvir plus asunaprevir) was approved for use in Japanese patients with HCV infection in July 2014. Therefore, high rates of sustained virological response (SVR) have been achieved in patients with chronic HCV infection^13–16^. Recently, DAAs have been introduced as an easy and safe antiviral oral therapy for HCV infection^16^.

In Japan, viral hepatitis remains the leading cause of HCC; however, the decrease in the prevalence of HCV-related HCC has changed the distribution of the disease etiology^7^. Specifically, while the proportion of HCC cases with non-viral etiology continues to increase in Japan^17^, epidemiological trends in sex and age distribution of new HCC cases remain unclear. Therefore, this study aimed to examine the epidemiological trends in HCC incident cases in Japan over the past 24 years (1996–2019).

## Results

A total of 20,547 patients were newly diagnosed with HCC during 1996–2019 (24 years, Table 1). The number of new HCC cases in the first, second, third, and fourth quarters was 4311 (21.0%), 5505 (26.8%), 5776 (28.1%), and 4955 (24.1%), respectively (Tables 2, 3, 4).

**Table 1:** Overall patient characteristics (*n* = 20,547).

Characteristics	Value
Sex (male/female)	14,020 (68.2%)/6527 (31.8%)
Age (years)	68.6 ± 10.1
Etiology (HBV/HBV + HCV/HCV/non-BC)	2997 (14.6%)/187 (0.9%)/12,019 (58.5%)/5344 (26.0%)

Results are expressed as the number (%) or the mean ± standard deviation.

Non BC=both hepatitis B surface antigen and HCV-antibody negative.

### Sex

**Figure 1:**
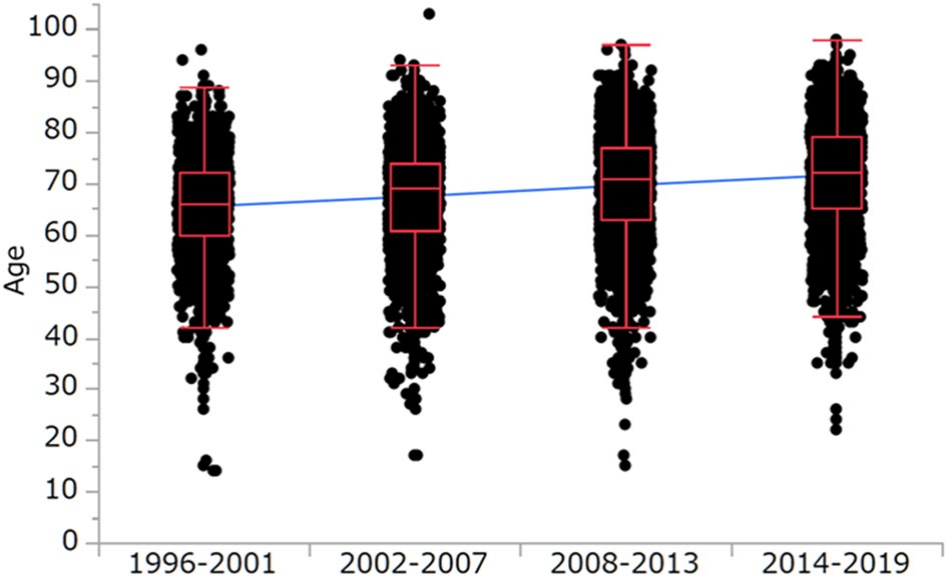
Age distribution of new hepatocellular carcinoma cases. 65.4 ± 9.2 years during 1996–2001 to 67.4 ± 9.8 years during 2002–2007, *P* < 0.0001; 67.4 ± 9.8 years during 2002–2007 to 69.5 ± 10.1 years during 2008–2013, *P* < 0.0001; and 69.5 ± 10.1 years during 2008–2013 to 71.5 ± 10.1 years during 2014–2019, *P* < 0.0001, respectively (mean ± standard deviation).

Overall, 14,020 (68.2%) and 6527 (31.8%) patients were males and females, respectively (Table 1). The number of cases in men and women per quarter were 3048 (70.7%) and 1263 (29.3%); 3732 (67.8%) and 1773 (32.2%); 3842 (66.5%) and 1934 (33.5%); 3398 (68.6%) and 1557 (31.4%), respectively (Table 2, Supplementary Fig. S1, S2). There were more female patients in the second quarter than in the first (*P* = 0.0020, Table 2); concurrently, there were more male patients in the fourth quarter than in the third (*P* = 0.0231, Table 2). Therefore, there was no noticeable change in the sex distribution of HCC incident cases throughout the study period (Fig. 1).

**Table 2:** Sex distribution of new-onset hepatocellular carcinoma cases in each quarter.

Sex	First quarter (1996–2001) *n* = 4311	Second quarter (2002–2007) *n* = 5505	Third quarter (2008–2013) *n* = 5776	Fourth quarter (2014–2019) *n* = 4955
Male *n* = 14,020 (68.2%)	3048 (70.7%)	3732 (67.8%)	3842 (66.5%)	3398 (68.6%)
Female *n* = 6527 (31.8%)	1263 (29.3%)	1773 (32.2%)	1934 (33.5%)	1557 (31.4%)
*P*-value	-	*P* = 0.0020 (vs. First quarter)	*P* = 0.1491 (vs. Second quarter)	*P* = 0.0231 (vs. Third quarter)

Data are expressed as counts (%).

### Age

Overall, the patients’ mean (± standard deviation, SD) age at diagnosis was 68.6 ± 10.1 years (Table 1). The numbers of HCC cases in patients < 60 years, 60–69 years, 70–79 years, and ≥ 80 years were 3711 (18.1%), 6652 (32.4%), 7448 (36.2%), and 2736 (13.3%), respectively (Table 3, Supplementary Fig. S3, S4). The mean ages (± SD) of the patients diagnosed with HCC in the first, second, third, and fourth quarters were 65.4 ± 9.2, 67.4 ± 9.8, 69.5 ± 10.1, and 71.5 ± 10.1 years, respectively, showing an increase over time (*P* < 0.0001, *P* < 0.0001, and *P* < 0.0001, respectively, Table 3, Fig. 1).

**Table 3:** Age distribution of new hepatocellular carcinoma cases in each quarter.

Age	First quarter (1996–2001) *n* = 4311	Second quarter (2002–2007) *n* = 5505	Third quarter (2008–2013) *n* = 5776	Fourth quarter (2014–2019) *n* = 4955
< 60 years *n* = 3711 (18.1%)	994 (23.0%)	1181 (21.5%)	955 (16.5%)	581 (11.7%)
60–69 years *n* = 6652 (32.4%)	1839 (42.7%)	1697 (30.8%)	1690 (29.3%)	1426 (28.8%)
70–79 years *n* = 7448 (36.2%)	1292 (30.0%)	2179 (39.6%)	2227 (38.6%)	1750 (35.3%)
≥ 80 years *n* = 2736 (13.3%)	186 (4.3%)	448 (8.1%)	904 (15.6%)	1198 (24.2%)
Mean	65.4 ± 9.2	67.4 ± 9.8	69.5 ± 10.1	71.5 ± 10.1
*P*-value	-	*P* < 0.0001 (vs. First quarter)	*P* < 0.0001 (vs. Second quarter)	*P* < 0.0001 (vs. Third quarter)

Data are presented as counts (%) or mean ± standard deviation.

### Etiology

The HCC diagnosis was associated with HBV, HBV + HCV, HCV, and non-BC exposure in 2997 (14.6%), 187 (0.9%), 12,019 (58.5%), and 5344 (26.0%) cases, respectively (Table 1). There were 579 (13.4%), 853 (15.5%), 889 (15.4%), and 676 (13.7%) new HCC cases associated with HBV in the first, second, third, and fourth quarters, respectively, showing no change in the overall incident cases over time (Table 4, Supplementary Fig. S5, S6). The corresponding values for HCV were 3147 (73.0%), 3636 (66.0%), 3233 (56.0%), and 2003 (40.4%) cases, respectively, showing a decrease over time (*P* < 0.0001, *P* < 0.0001, and *P* < 0.0001, respectively, Table 4). Finally, the corresponding values for non-BC were 518 (12.0%), 951 (17.3%), 1615 (27.9%), and 2260 (45.6%) cases, respectively, showing an increase over time (*P* < 0.0001, *P* < 0.0001, and *P* < 0.0001, respectively, Table 4).

**Table 4:** Distribution of disease etiology among new hepatocellular carcinoma cases in each quarter.

Etiology	First quarter (1996–2001) *n* = 4311	Second quarter (2002–2007) *n* = 5505	Third quarter (2008–2013) *n* = 5776	Fourth quarter (2014–2019) *n* = 4955
HBV *n* = 2997 (14.6%)	579 (13.4%)	853 (15.5%)	889 (15.4%)	676 (13.7%)
HBV + HCV *n* = 187 (0.9%)	67 (1.6%)	65 (1.2%)	39 (0.7%)	16 (0.3%)
HCV *n* = 12,019 (58.5%)	3147 (73.0%)	3636 (66.0%)	3233 (56.0%)	2003 (40.4%)
Non-BC *n* = 5344 (26.0%)	518 (12.0%)	951 (17.3%)	1615 (27.9%)	2260 (45.6%)
*P*-value	-	*P* < 0.0001 (vs. First quarter)	*P* < 0.0001 (vs. Second quarter)	*P* < 0.0001 (vs. Third quarter)

Data are presented as counts (%).

Non BC=both hepatitis B surface antigen and HCV-antibody negative.

### Correlation between age and etiology

**Figure 2:**
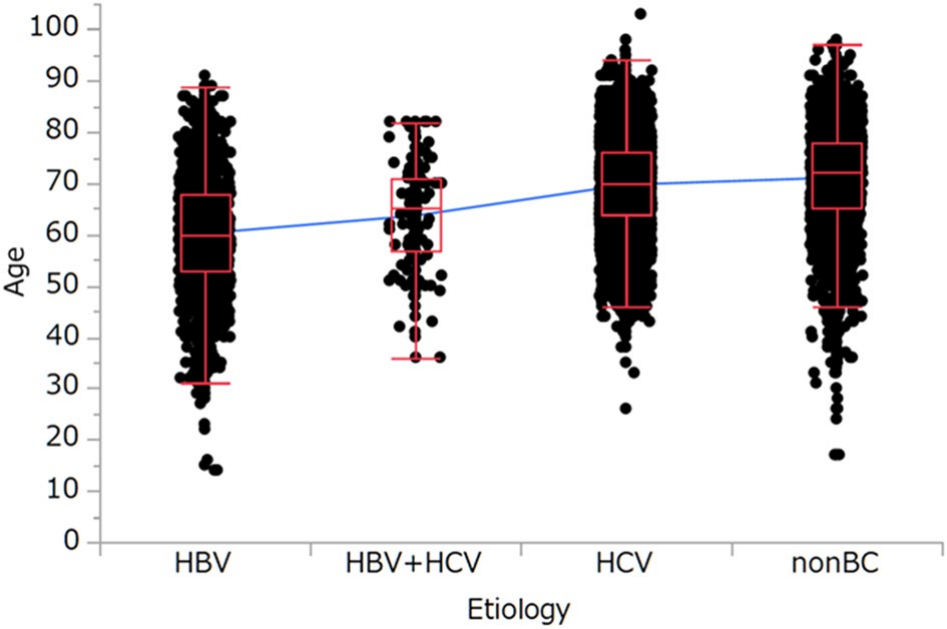
Correlation between patient age and disease etiology. 60.2 ± 10.9 years in HBV to 63.6 ± 10.0 years in HBV + HCV, *P* < 0.0001; 63.6 ± 10.0 years in HBV + HCV to 69.7 ± 8.8 years in HCV, *P* < 0.0001; 69.7 ± 8.8 years in HCV to 71.0 ± 9.9 years in non-BC, *P* < 0.0001, respectively (mean ± standard deviation).

The mean ages (± SD) of the patients with HCC associated with HBV, HBV + HBC, HCV, and non-BC were 60.2 ± 10.9, 63.6 ± 10.0, 69.7 ± 8.8, and 71.0 ± 9.9 years, respectively (Fig. 2). There was a significant association between patient age and disease etiology (*P* < 0.0001, *P* < 0.0001, and *P* < 0.0001, respectively, Fig. 2).

## Discussion

In the present study, we examined epidemiological trends in HCC incident cases, including the distribution of sex, age, and disease etiology over 24 years. Although there was no noticeable change in the sex distribution of HCC incident cases throughout the study period, the average age of newly diagnosed HCC patients increased along the quarters. The number of HCV-associated cases decreased over time, while non-BC-associated cases increased over time. Moreover, there was a significant association between patient age and disease etiology.

In July 2014, DAAs were approved for Japanese patients with HCV infection. The development of DAAs has made it easier to treat HCV infections in the elderly and cirrhotic patients, and not only at specialized high-volume centers but also at general practice clinics^16^. As a result, the eradication of HCV infection has been reported to reduce HCC risk^18^. In the present study, the number of new HCC cases increased from the first to the third quarter (1996–2013), decreasing, after that, from the third to the fourth quarter (2014–2019). This relative reduction in HCC cases observed in the fourth quarter (2014–2019) may be associated with improved management of HCV infections with DAAs.

HCC incidence is two–threefold higher among men than women in most regions worldwide, while liver cancer ranks fifth and second in the global number of cases and associated deaths among men, respectively^1^. Meanwhile, liver cancer incidence is forecasted to decrease among men in Japan and China and women in Japan and Denmark^2^. In the present study, there was no association between sex and HCC incident cases over time. Therefore, while the incident case of HCC is expected to decrease among both men and women in Japan, a sex gap in the burden of this disease remains.

In the present study, patient age among newly diagnosed HCC cases increased over time. Meanwhile, the number of HCV-associated HCC cases decreased over time, in contrast to non-BC-associated cases, which increased over time. Additionally, we observed an association between patients’ age and disease etiology. Overall, HBV-associated HCC patients tended to be younger (mean age ± SD at onset 60.2 ± 10.9 years) than HCV-associated HCC patients (mean age ± SD at onset 69.7 ± 8.8 years). Therefore, it was predicted that the age of new-onset HCC patients would decrease by the decrease in new-onset HCV-associated HCC cases; however, the age of new-onset HCC patients has instead increased. This is because in the present study, the proportion of non-BC-associated HCC cases (mean age ± SD at onset 71.0 ± 9.9 years) was higher than that of HCV-associated HCC cases (mean age ± SD at onset 69.7 ± 8.8 years), and it continued to increase. In the future, as the incident case of non-BC-associated HCC increases, the age of the incident case of HCC will also increase.

The use of DAAs in HCV-infected patients has been shown to lower the risk of liver-related events, including HCC^19^. However, despite an SVR of > 95%, the HCC risk in DAA-treated HCV-infected patients—with advanced fibrosis or cirrhosis—was shown to remain between 0.3 and 1.8% per year^20,21^. The current European Association for the Study of the Liver (EASL) and American Association for the Study of Liver Diseases (AASLD) guidelines recommend lifetime surveillance of HCV-cured patients with cirrhosis^22,23^. Identifying clinical and molecular markers associated with HCC risk among these patients may improve the treatment effectiveness and resource allocation^22,23^. Studies on epidemiology and precision medicine may help inform, refine, and customize clinical guidelines for disease surveillance in this context^24^.

In the present study, the average age of patients newly diagnosed with HCC and those newly diagnosed with non-BC-associated HCC increased over time, while the patients newly diagnosed with HCV-associated HCC decreased over time. The increasing age of patients newly diagnosed with non-BC-associated HCC is likely to become a public health concern in the future. Recent studies have reported an association between metabolic syndrome (diabetes and obesity), excessive alcohol consumption (alcoholic fatty liver disease), and high-calorie intake (nonalcoholic fatty liver disease), and HCC risk in countries characterized by Westernized sedentary lifestyles^25^. A detailed understanding of the relevant risk factors is paramount for improving HCC screening, diagnosis, management, and prevention strategies^25^.

This study had some limitations. Firstly, while the hepatic reserve effect in liver carcinogenesis was known, in the present study, we focused mainly on three factors, sex, age, and disease etiology. Therefore, a multicenter study with additional clinical information is suggested in the future. Secondly, we did not investigate the size or number of HCC incident cases, namely, its stages. Future studies investigating the correlation between the HCC stage and sex, age, or disease etiology are required.

In conclusion, the present study suggests that HCC incident cases in the elderly and due to non-BC tended to increase, while the incident cases of HCV-associated HCC tended to decrease between quarters. In countries where HCV infection is likely the predominant cause of HCC, like Japan, similar trends in HCC incident case are anticipated in the future.

## Methods

This prospective study was approved by the Ethics Committee of the National Hospital Organization Nagasaki Medical Center (no. 2020053) and was conducted according to the guidelines of the 1975 Declaration of Helsinki^26^.

We included only those newly diagnosed HCC patients in this study diagnosed at one of the 19 participating institutions of the Liver Cancer Study Group of Kyushu between 1996 and 2019 (24 years). The distribution of factors such as sex, age, and disease etiology was examined among the new HCC cases. The study period was divided into four quarters of six years each: 1996–2001(first quarter), 2002–2007 (second quarter), 2008–2013 (third quarter), and 2014–2019 (fourth quarter).

### Diagnosis

We diagnosed HCC by measuring alpha-fetoprotein and des-gamma-carboxy prothrombin serum levels and via imaging techniques, including ultrasonography, contrast-enhanced computerized tomography, magnetic resonance, and/or tumor biopsies.

### Etiology

The etiology of HCC was categorized as follows: HBsAg positive and HCV-antibody negative (HBV), both HBsAg and HCV-antibody positive (HBV + HCV), HBsAg negative and HCV-antibody positive (HCV), and both HBsAg and HCV-antibody negative (non-BC).

### Statistical analysis

Inter-quarter differences in sex and disease etiology frequencies were calculated using the chi-square test, and age differences were examined using the one-way analysis of variance (ANOVA) and post-hoc analysis (Bonferroni) methods. All the statistical analyses were performed using JMP software version 15 (SAS Institute, Inc., Cary, NC, USA). *P*-values of < 0.05 were considered significantly different.

### Ethical approval

All procedures performed in studies involving human participants were in accordance with the ethical standards of the institutional and/or national research committee (Ethics Committee of the National Hospital Organization Nagasaki Medical Center, no. 2020053) and with the 1964 Helsinki declaration and its later amendments or comparable ethical standards.

### Informed consent

Informed consent was obtained from all individual participants included in the study.

## Supplementary Information

Supplementary Information 1.

Supplementary Information 2.

Supplementary Information 3.

Supplementary Information 4.

Supplementary Information 5.

Supplementary Information 6.

Supplementary Information 7.

## Data Availability

The data that support the findings of this study are available from the corresponding author, HY, on reasonable request.
