# Supplementary figures and images for: Trends in hepatocellular carcinoma incident cases in Japan between 1996 and 2019
Source: Sci Rep. 2022 Jan 27;12:1517. doi: 10.1038/s41598-022-05444-z (PMC8795252; doi:10.1038/s41598-022-05444-z)

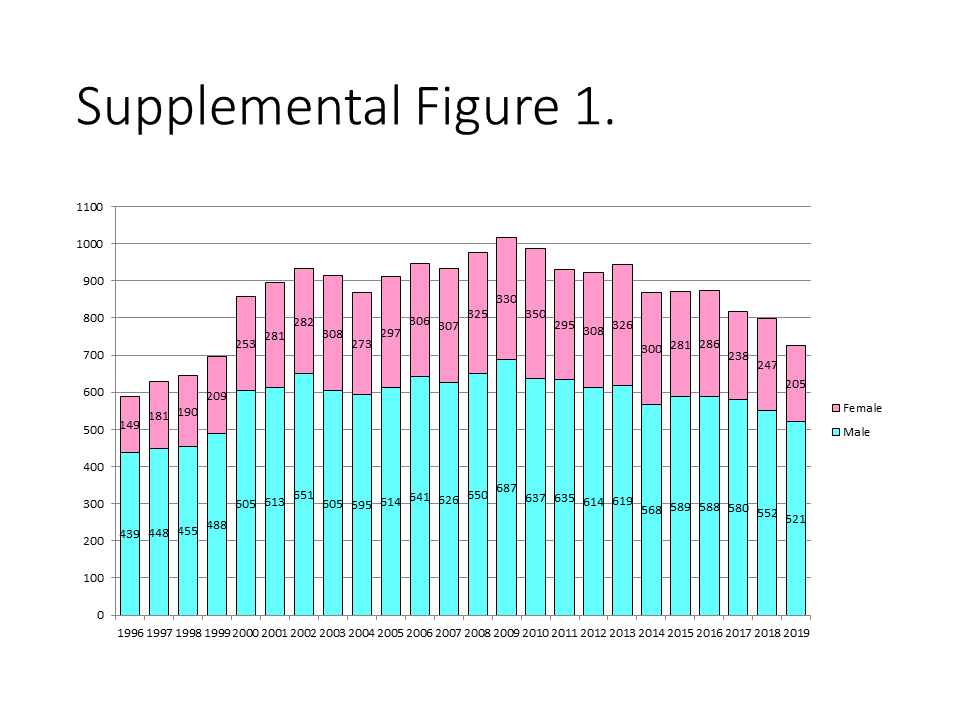

Supplement: Supplementary file 1 — Supplementary Information 1. [file 41598_2022_5444_MOESM1_ESM.tif]

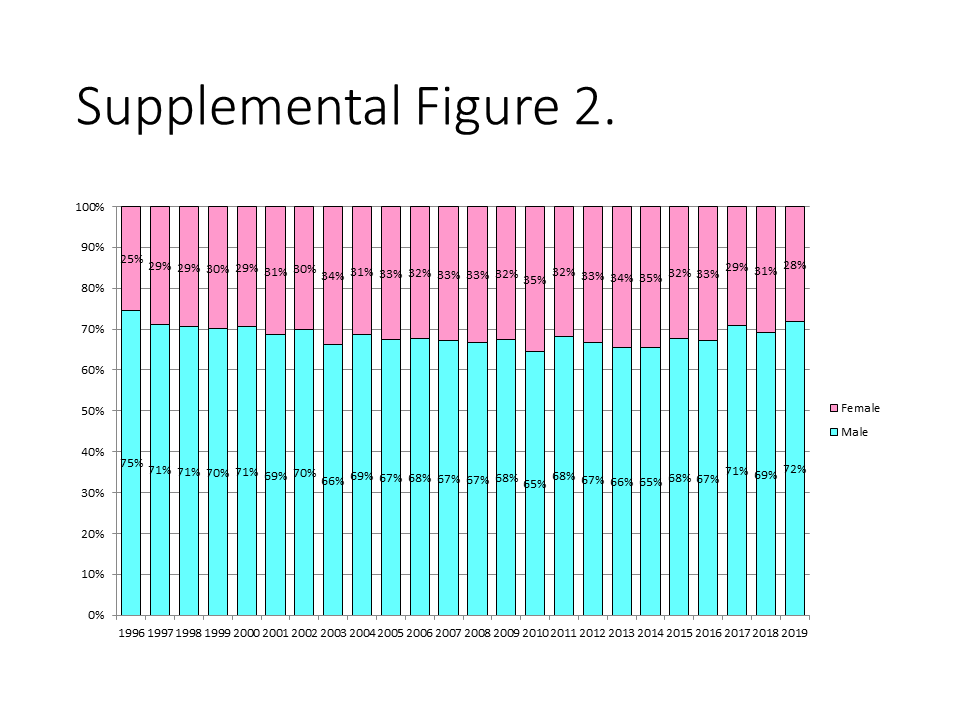

Supplement: Supplementary file 2 — Supplementary Information 2. [file 41598_2022_5444_MOESM2_ESM.tif]

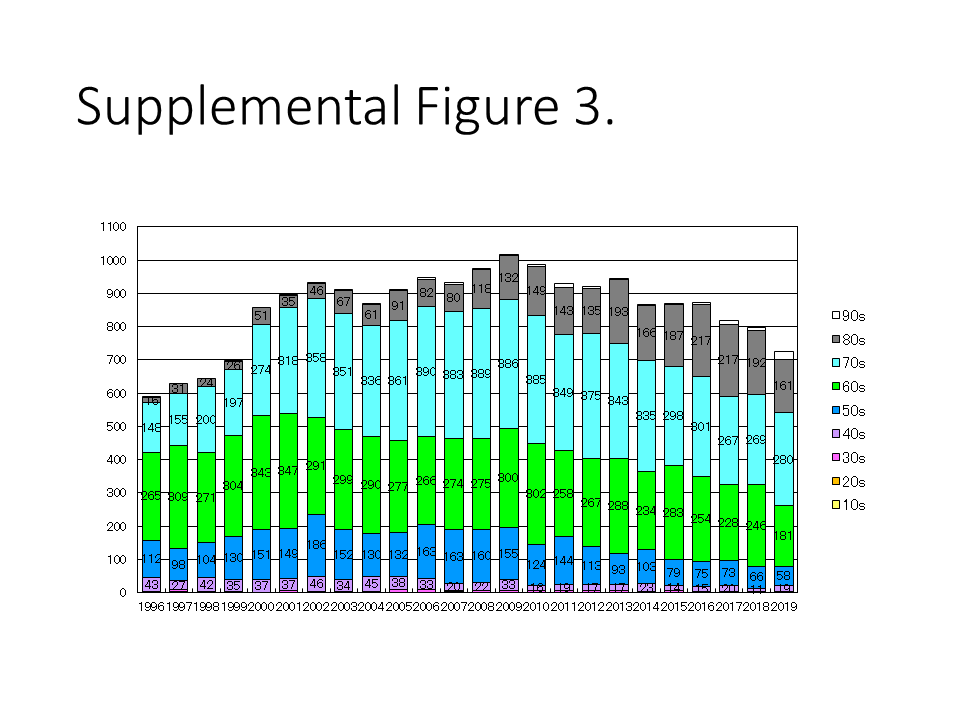

Supplement: Supplementary file 3 — Supplementary Information 3. [file 41598_2022_5444_MOESM3_ESM.tif]

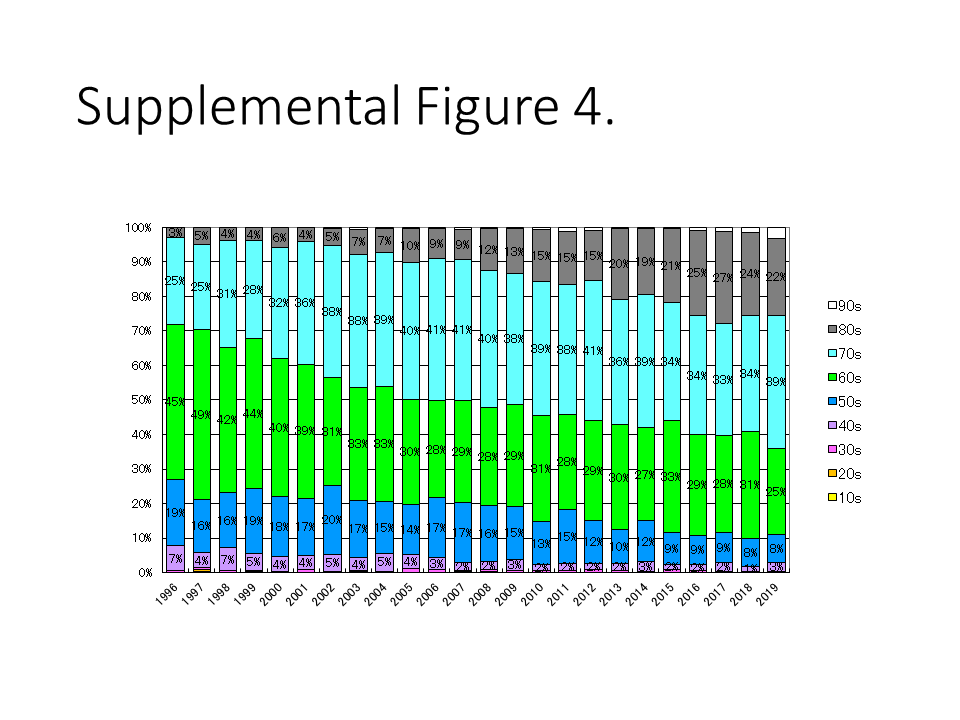

Supplement: Supplementary file 4 — Supplementary Information 4. [file 41598_2022_5444_MOESM4_ESM.tif]

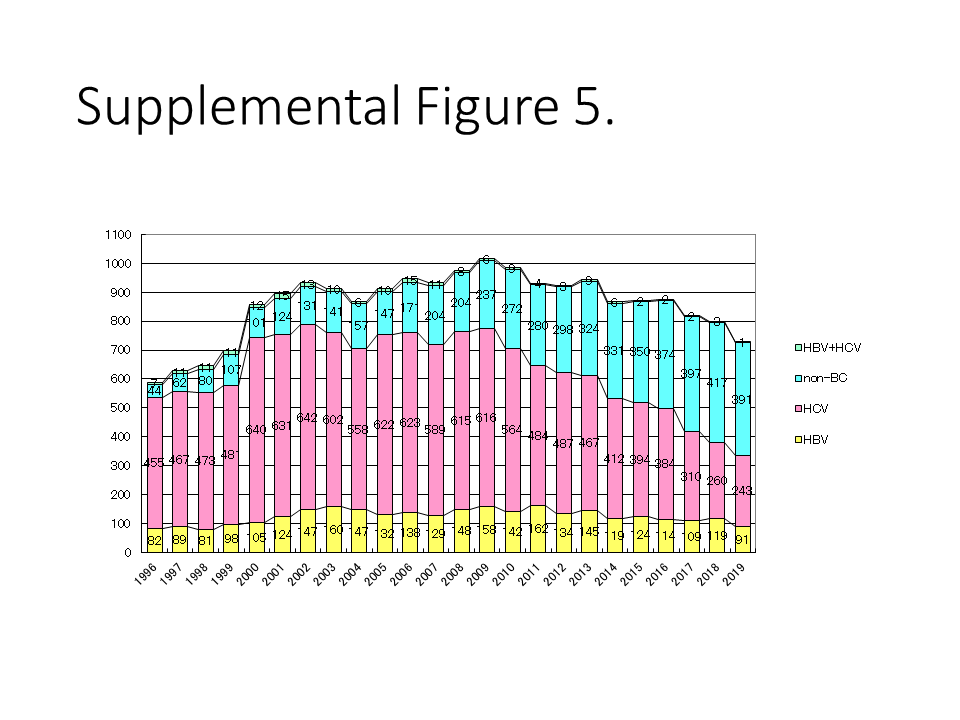

Supplement: Supplementary file 5 — Supplementary Information 5. [file 41598_2022_5444_MOESM5_ESM.tif]

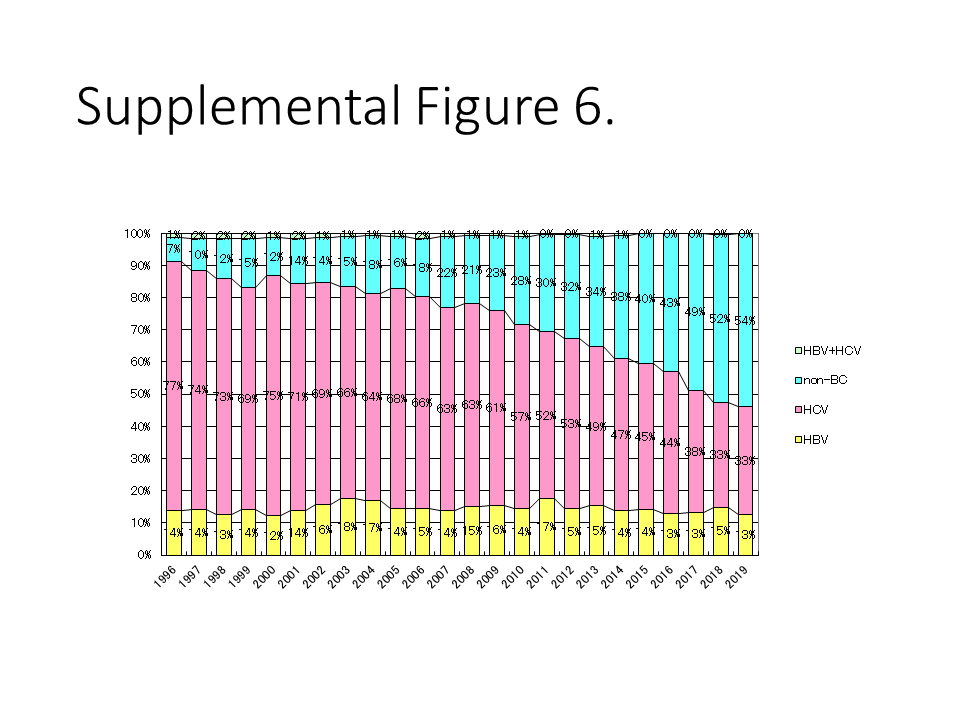

Supplement: Supplementary file 6 — Supplementary Information 6. [file 41598_2022_5444_MOESM6_ESM.tif]
