# Supplementary material for: Trends in hepatocellular carcinoma incident cases in Japan between 1996 and 2019
Source: Sci Rep. 2022 Jan 27;12:1517. doi: 10.1038/s41598-022-05444-z (PMC8795252; doi:10.1038/s41598-022-05444-z)
Supplement: Supplementary file 7 — Supplementary Information 7. [file 41598_2022_5444_MOESM7_ESM.docx]

**Supplementary Figure Legends**

**Supplementary Fig. S1, S2 Sex distribution of new hepatocellular carcinoma cases.**

**Fig. 1** Count

**Fig. 2** Ratio

**Supplementary Fig. S3, S4 Age distribution of new hepatocellular carcinoma cases.**

**Fig. 3** Count

**Fig. 4** Ratio

**Supplementary Fig. S5, S6 Disease etiology distribution of new hepatocellular carcinoma cases.**

**Fig. 5** Count

**Fig. 6** Ratio
